# Leukotriene Receptor Antagonists for the Prevention and Treatment of Capsular Contracture: A Systematic Review and Meta-analysis

**DOI:** 10.7759/cureus.86566

**Published:** 2025-06-22

**Authors:** Omer Bin Abdul Aziz, Meezan Jalil, Bilal Ahmad, Aamir Hussain, Muhammad Awais Mughal

**Affiliations:** 1 Surgery, Qassim Armed Forces Hospital, Buraydah, SAU; 2 Surgery, Al Kharj Armed Forces Hospital, Al-Kharj, SAU; 3 Endocrinology and Diabetes, Qassim Armed Forces Hospital, Buraydah, SAU; 4 Surgery, Prince Mansour Military Hospital, Taif, SAU

**Keywords:** capsular contracture, leukotriene receptor antagonist, management, montelukast, zafirlukast

## Abstract

Capsular contracture (CC) is a common complication following breast implant surgery, characterized by excessive fibrous tissue formation around the implant. Leukotriene receptor antagonists (LRAs), such as montelukast and zafirlukast, have been investigated for their anti-inflammatory and anti-fibrotic properties as potential preventive and therapeutic agents for CC. However, findings remain inconclusive.

A systematic literature search was conducted, and studies involving human subjects that utilized the Baker scale for CC assessment and were published in English were included. Relevant studies were reviewed, and a meta-analysis was performed.

The pooled risk difference (RD) was -0.29, with a standard error (SE) of 0.10 and a corresponding 95% CI of -0.48 to -0.09. This finding was statistically significant (Z-value = -2.81), suggesting that LRAs are effective in the prevention and treatment of CC. Subgroup analysis demonstrated that zafirlukast had a significant effect in reducing CC (RD = -0.40, SE = 0.10, 95% CI -0.60 to -0.20, Z-value = -3.96, p = 0.00).

This systematic review and meta-analysis indicate that LRAs, particularly zafirlukast, are effective in reducing the severity and recurrence of CC, especially in its early stages. Further high-quality clinical trials are warranted to establish standardized guidelines for the use of LRAs in the management of CC.

## Introduction and background

Capsular contracture (CC) is a pathological fibrotic response to breast implants, characterized by progressive thickening, hardening, or deformation of the fibrous capsule surrounding the implant [1]. It remains one of the most common complications of implant-based breast surgery, affecting 15%-45% of patients after augmentation mammoplasty [2]. In post-mastectomy breast reconstruction, CC rates range from 2.8% to 15.9% [3]. Radiotherapy substantially increases the risk, with reported rates 17%-86% [4-6]. However, significant heterogeneity among studies limits the ability to derive a precise incidence estimate. Revision surgeries for CC are associated with substantial healthcare costs, occurring in 28%-50% of cases [7]. The current gold-standard treatment (total capsulectomy and implant replacement) is reported to carry high surgical risks and high recurrence rates [8]. Although the exact etiology of CC remains unclear, it is believed to involve an inflammatory process. Emerging evidence links bacterial endotoxins to macrophage-driven immune responses that amplify fibrotic remodeling [9]. Moreover, cytokines such as TGF-β and IL-6 play important roles in fibroblast activation and extracellular matrix deposition [10]. Leukotrienes are known to exacerbate these pathways, providing a rationale for the use of LRAs as potential therapeutic agents. The development of effective non-surgical interventions for CC could help reduce psychological distress, minimize delays in return to work, and alleviate the broader socioeconomic burden associated with this condition.

## Review

Methodology

A systematic literature search was conducted to identify all relevant articles published on the use of LRAs in the prevention and treatment of CC. Eligibility criteria were applied to screen and select studies for inclusion. Eligible studies were then included in the systematic review. The selection process adhered to the Preferred Reporting Items for Systematic Reviews and Meta-Analyses (PRISMA) guidelines.

Online Information and Databases Search

On the basis of a predefined eligibility criteria, a systematic literature search was performed using the online databases PubMed Central (PMC) and PubMed/Medline to identify all relevant studies.

The search strategy used was as follows: (Leukotriene receptor antagonist OR Leukotriene receptor antagonists OR Leukotriene antagonist OR Leukotriene antagonists OR AntiLeukotriene OR Antileukotrienes OR Leukotriene receptor inhibitor OR Leukotriene receptor inhibitors OR Leukotriene Inhibitors OR LRA OR LRTA OR Accolate OR Singulair OR Zafirlukast OR Montelukast) AND (Capsular Contracture OR Implant Capsular Contracture OR Contracture of the capsule) AND (Mammaplasty OR Mammaplasties OR Mammoplasty OR mammoplasties OR Breast augmentation OR Breast reconstruction OR Implant based breast reconstruction OR IBBR). All searches were conducted using both Medical Subject Headings (MeSH) and free-text terms

Eligibility Criteria

The inclusion criteria were as follows: (1) studies involving human subjects who underwent implant-based surgical procedures, including breast augmentation, mammoplasty, mastopexy with prosthesis, implant-based breast reconstruction, or revision surgery; (2) studies using LRAs for the prevention or treatment of CC; (3) studies comparing patients who underwent implant-based breast surgery without receiving LRAs; and (4) studies assessing CC using the modified Baker scale [11] (Table 1). Patients with Grade II or higher on the Baker scale were considered to have CC. Patients presenting with Grade IB were not considered candidates for prophylactic LRA therapy. An improvement of 0.5 or more on Baker scale was regarded as an effective treatment outcome. Conversely, if the Baker scale grade did not decrease or increased following therapy, the treatment was considered a failure. In cases where prophylactic therapy maintained the Baker scale grade at Grade I or lower, it was classified as effective prevention. The additional inclusion criteria were (5) randomized controlled trials (RCTs), non-randomized trials, cohorts, and case series with either prospective or retrospective designs involving human subjects and (6) studies published in English or those with results available in English.

**Table 1:** Modified Baker classification Source: [11].

S. no.	Class	Description
1	Class IA	Absolutely natural; cannot tell breast was reconstructed
2	Class IB	Soft, but the device is detectable by physical examination or inspection because of the mastectomy
3	Class II	Mildly firm reconstructed breast with a device that may be visible and detectable by physical examination
4	Class IIB	Moderately firm reconstructed breast with a device that is really detectable, but the result is acceptable and does not require operative intervention
5	Class III	Moderately firm reconstructed breast with a device that is readily detectable and requires operative intervention
6	Class IV	Severe capsular contracture with an unacceptable aesthetic outcome and/or significant patient symptoms that require operative intervention

The exclusion criteria were as follows: (1) review articles, meta-analyses, in vitro and experimental animal studies, duplicate publications, systematic reviews, case reports, letters, and commentaries; (2) studies that did not use Baker classification for assessing CC; (3) studies published in languages other than English; and (4) studies with incomplete data or whose full-text article could not be retrieved.

Study Selection and Data Extraction

The search results were screened for duplicates, which were subsequently removed. Titles and abstracts of the remaining studies were assessed for relevance, and non-relevant studies were excluded. Full texts of the potentially eligible studies were then retrieved and reviewed. Inclusion and exclusion criteria were applied to identify eligible studies. Data extraction was performed independently by three reviewers (O.B.A.A, M.J., and B.A.), with discrepancies resolved by a fourth reviewer (M.A.M). Extracted data included first author, year of publication, study design, patient age, type of leukotriene receptor antagonist used, type of implant, treatment duration and follow-up, surgical details including incision and pocket placement, and any reported adverse effects of treatment.

Quality Assessment of Included Studies

The quality of the included non-randomized trials and cohort studies was assessed using the Newcastle-Ottawa Scale (NOS) [12].

For each study, the risk difference (RD) was calculated along with its standard error (ER) and corresponding 95% CI. A p-value < 0.05 was considered statistically significant. Heterogeneity among studies was assessed using the chi-squared test, with its impact quantified by the I² statistic. An I² value > 75% was considered indicative of high heterogeneity, and a p-value < 0.10 denoted statistically significant heterogeneity. In cases of significant heterogeneity (I² > 50% or p < 0.10), a random-effects model was applied to compute the pooled RD and 95% CI. Funnel plots were used to evaluate potential publication bias. Additionally, a subgroup analysis was conducted to determine the specific effects of each LRA on the outcome.

Results

Selection of Studies Included in the Review

**Figure 1:**
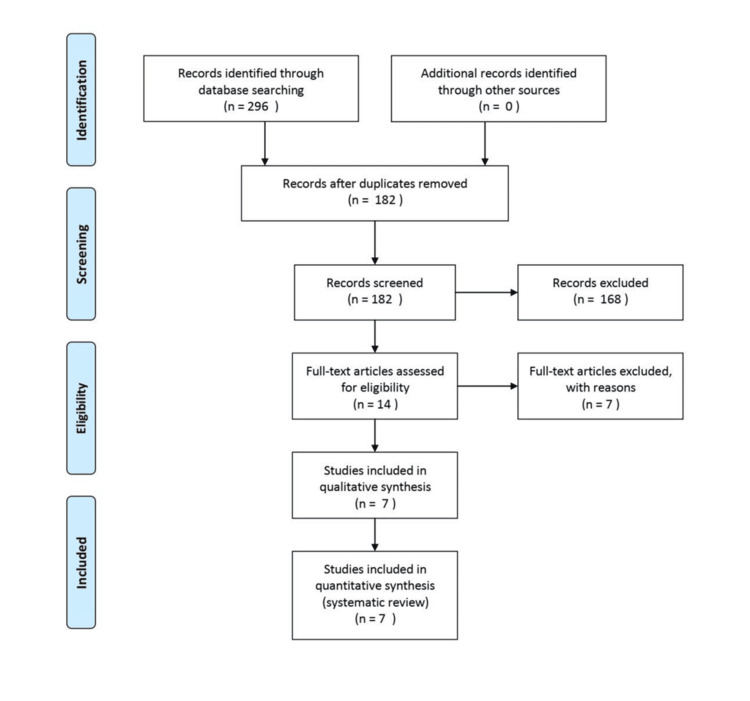
PRISMA flow diagram of the study selection process

The initial database search was conducted in February 2024, with an updated search completed in January 2025. A total of 296 studies were initially identified. After removing duplicate studies, 182 articles remained. Based on title and abstract screening, 168 studies were excluded due to irrelevance, being review articles, non-English publications, animal studies, or meta-analyses. The full texts of the remaining 14 articles were then reviewed. Of these, seven publications were excluded for the following reasons: two did not evaluate CC using the Baker scale, one was a letter to the editor, one was a commentary, one was a “review of practices,” one was a hypothesis-generating study, and one was a case series of five cases without statistical analysis. Finally, seven studies met the inclusion criteria and were included in the systematic review [13-19]. Figure 1 presents the PRISMA flow diagram outlining the study selection process.

Study Characteristics

Of the seven eligible studies, three were prospective in design and four were retrospective. None of the included studies were randomized controlled trials (RCTs). A total of 1324 women were enrolled across studies, with sample sizes ranging from 19 to as 1122 participants. The follow-up periods varied considerably, ranging from 1 month to 36 months, with differences in follow-up intervals and durations. Treatment duration was typically three months in most studies, except for one study in which treatment lasted between three and six months [16]. Montelukast was administered at a dosage of 10 mg orally once daily, while zafirlukast was given at 20 mg orally twice daily (Table 2).

**Table 2:** Individual study characteristics ROS: retrospective observational study, POS: prospective observational study, non-RCT: non-randomized controlled trial.

Author (year)	Age in years	Study design	Treatment and duration (in months)	Follow-up duration (in months)
Graf et al. (2015) [13]	18-55 (33)	ROS	Montelukast 10 mg p.o. o.d. 3 months	24
Bresnick (2017) [14]	22-60 (mean not given)	ROS	Montelukast 10 mg p.o. o.d. or zafirlukast 20 mg p.o. b.i.d 3 months	12
Huang and Handel (2010) [15]	44.2 (no range given)	ROS	Montelukast 10 mg p.o. o.d. 3 months	18-61
Reid et al. (2005) [16]	18-52 (34.8)	POS	Zafirlukast 20 mg p.o. b.i.d 3-6 months	6-29
Procikieviez and Procikieviez (2024) [17]	45 + 18	ROS	Montelukast 10 mg p.o. o.d.	12-18
Scuderi et al. (2006) [18]	25-54 (36 years 9 months)	POS	Zafirlukast 20 mg p.o. b.i.d 6 months	-
Lille and Jacoby (2018) [19]	-	Non-RCT	Montelukast 3 months	11 + 4.5

The included studies demonstrated considerable variability in surgical procedures, implant types and sizes, prosthesis materials, and implant placement, as summarized in Table 3. This heterogeneity affects the overall quality and comparability of the systematic review. While all the studies reported the type and texture of the implant used, Bresnick [14] and Procikieviez and Procikieviez [17] did not specify the size, volume, or shape of the implants used in their respective study populations. A range of surgical procedures was performed across studies, with some procedures included in one study and excluded in another. Similarly, the type of implant pocket also varied among the included studies

**Table 3:** Details of prostheses and procedures

Author (year)	Implant material	Implant size (cc)	Implant placement	Surgical procedure	Incision
Graf et al. (2015) [13]	Silicone textured	150-495	Submuscular, subglandular, subfascial	Breast augmentation, mastopexy with prosthesis, prostheses exchange	Inframammary, periareolar, axillary, inverted T
Bresnick (2017) [14]	Silicone smooth	-	Dual plane	Breast augmentation	Periareolar, inframammary
Huang and Handel (2010) [15]	Saline or silicone smooth	250-700	Subpectoral, submammary	Primary augmentation, breast reconstruction, implant exchange, capsulotomy, capsulectomy	Periareolar, crescent mastopexy, Benelli, inframammary vertical mastopexy, Wise pattern
Reid et al. (2005) [16]	Saline smooth	230-430	Submuscular	Augmentation mammoplasty	-
Procikieviez and Procikieviez (2024) [17]	Silicone textured	-	Retropectoral, dual plane	Secondary breast augmentation	Periareolar, inframammary
Scuderi et al. (2006) [18]	Cohesive silicone, double-lumen silicone and saline, silicone textured	-	Submuscular	Breast augmentation, revision mammoplasty, breast reconstruction	Inframammary
Lille and Jacoby (2018) [19]	Saline smooth	-	Retromuscular	Breast augmentation/mastopexy	-

Risk of Bias Within Studies

The NOS score was used to assess the quality of the included studies. All studies achieved good scores of 7 or 8, indicating a low risk of bias, as summarized in Table 4.

**Table 4:** Newcastle-Ottawa Scale for evaluating the quality of non-randomized trials and cohorts Note: Asterisks represent "stars" of the Newcastle-Ottawa Scale (NOS), which refer to the points awarded to studies based on their quality across different domains.

Author (year)	Selection	Comparability	Outcome	Total	Quality
Graf et al. (2015) [13]	****	*	***	********	Good
Bresnick (2017) [14]	****	*	***	********	Good
Huang and Handel (2010) [15]	***	*	***	*******	Good
Reid et al. (2005) [16]	****	*	**	*******	Good
Procikieviez and Procikieviez (2024) [17]	****	*	***	********	Good
Scuderi et al. (2006) [18]	***	*	***	*******	Good
Lille and Jacoby (2018) [19]	***	*	***	*******	Good

Effects of LRA Treatment

Table 5 presents the numerical data for all breasts that that received or did not receive LRA therapy, either for prophylaxis or treatment of CC. It also lists the number of positive events in each group, that is, the incidence of CC in the untreated and prophylaxis groups and the lack of improvement in Baker scale grade in the treatment group.

**Table 5:** Control and experimental groups and positive events LRAs: leukotriene receptor antagonists.

Author (year)	Total breasts	Breasts treated with LRAs	Untreated breasts	Positive events in treated breasts	Positive events in untreated breasts
Graf et al. (2015) [13]	164	74	90	7	19
Bresnick (2017) [14]	1852	1288 (882 + 406)	564	34	28
Huang and Handel (2010) [15]	21 + 25	25	21	10	21
Reid et al. (2005) [16]	74 + 41	41	74	8	41
Procikieviez and Procikieviez (2024) [17]	128 (64 patients)	40 (20 patients)	88 (44 patients)	2 (1 patient)	14 (7 patients)
Scuderi et al. (2006) [18]	72	36	36	5	27
Lille and Jacoby (2018) [19]	72	37	35	0	4

**Figure 2:**
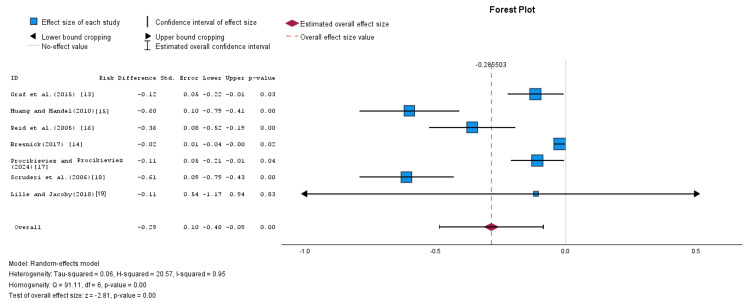
Forest plot summarizing the overall efficiency rates of LRAs in the prevention and treatment of CC (random-effects model) LRAs: leukotriene receptor antagonists, CC: capsular contracture. Source: [13-19].

This was further demonstrated using the RD metric. The overall RD was calculated by pooling all data from all seven studies using a random-effects model. The pooled RD was -0.29, with an SE of 0.10 and a corresponding 95% CI ranging from -0.48 to -0.09. The finding was statistically significant (Z-value = -2.81). However, heterogeneity among the studies was substantial, with an I² value of 95% (p = 0.00), as shown in Figure 2.

Risk of Bias in Studies

**Figure 3:**
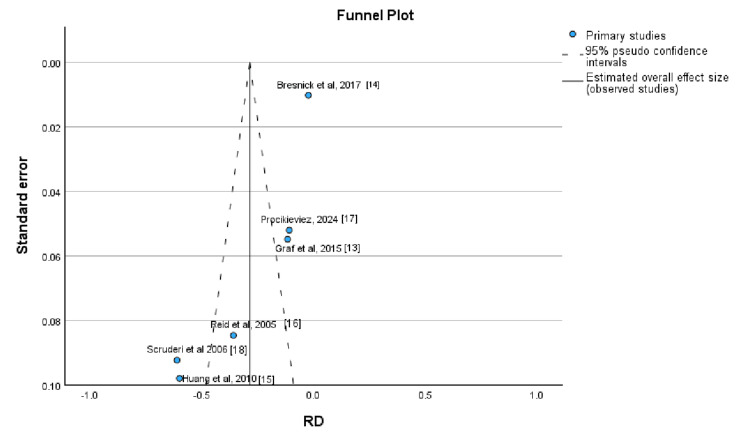
Funnel plot illustrating the assessment of publication bias among the included studies Source: [13-18].

Publication bias was assessed using a funnel plot, which demonstrated a tendency toward asymmetry, suggesting the possible presence of publication bias (Figure 3).

Subgroup Analysis

**Figure 4:**
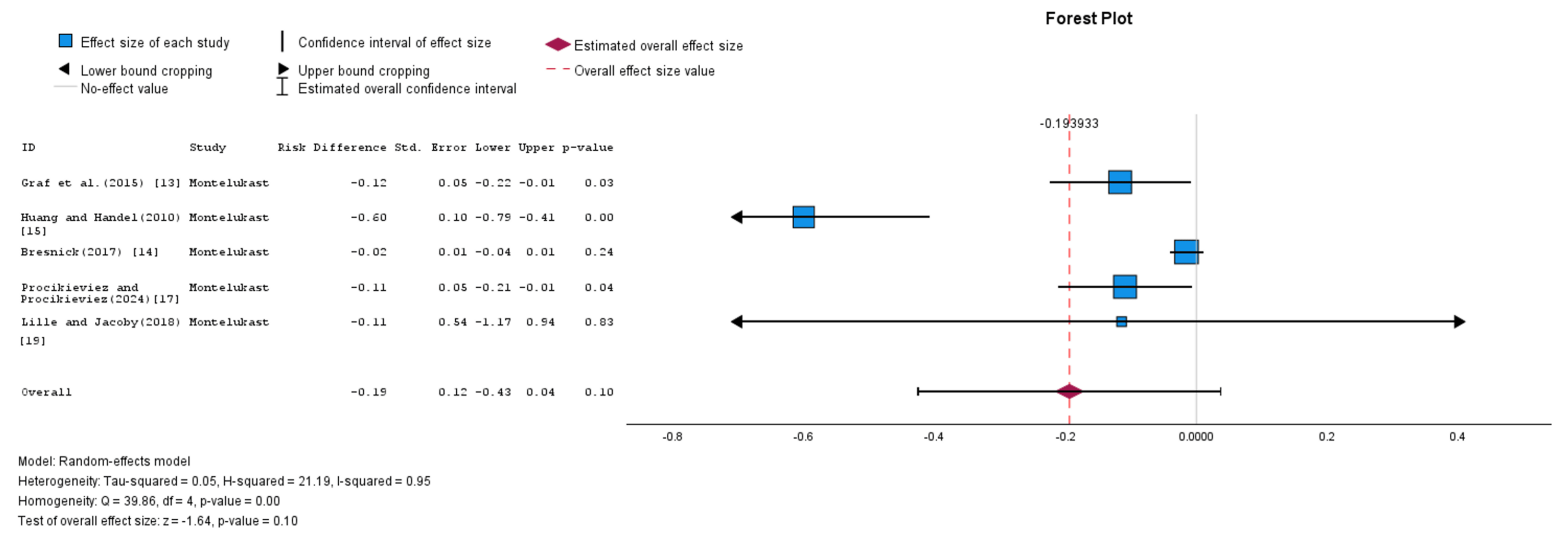
Forest plot illustrating the efficiency rates of montelukast in the prevention and treatment of CC (random-effects model) CC: capsular contracture. Source: [13-15,17,19].

**Figure 5:**
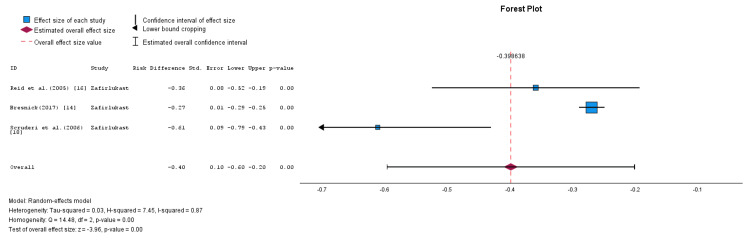
Forest plot showing the efficiency rates of zafirlukast in the prevention and treatment of CC (random-effects model) CC: capsular contracture. Source: [14,16,18].

A subgroup analysis was conducted based on the type of LRAs used in the studies. This subgroup analysis is illustrated in Figure 4 and Figure 5. The montelukast subgroup included five studies that utilized montelukast as the LRA of choice [13-15,17,19], and the pooled RD was calculated using a random-effects model, as shown in Figure 4. The pooled RD was -0.19, with an SE of 0.12, and a corresponding 95% CI ranging from -0.43 to 0.04 (p = 0.10). The finding was not statistically significant (Z-value = -1.64). The results showed that montelukast may not be effective in preventing or treating CC. The heterogeneity within this subgroup was high, with an I² value of 95%.

Using a random-effects model, the RD in the zafrilukast subgroup was calculated by pooling data from three studies that utilized zafirlukast as the LRA of choice [14,16,18], as shown in Figure 5. The pooled RD was -0.40, with an SE of 0.10, and a corresponding 95% CI ranging from -0.60 to -0.20. This result was statistically significant (Z-value = -3.96; p = 0.00). However, heterogeneity among the studies remained high, with an I² value of 87%. These findings support the potential clinical utility of zafirlukast in effectively managing CC.

Discussion

This systematic review and meta-analysis demonstrates that LRAs may be effective in the prevention and treatment of CC. The pooled RD was -0.29, with an SE of 0.10 and a 95% CI ranging from -0.49 to -0.09. This was statistically significant (Z-value = -2.81; p = 0.005), indicating that LRAs can be considered a viable option in the management of CC. Subgroup analysis revealed important distinctions between the two LRAs studied. Zafirlukast showed significant efficacy in preventing and treating CC, with patients in this subgroup demonstrating a 40% greater chance of improvement compared to controls (pooled RD = -0.40, 95% CI -0.60 to -0.20, Z = -3.96, p = 0.00). In contrast, montelukast, despite showing favorable outcomes in individual studies, did not yield statistically significant results in the pooled analysis (pooled RD = -0.19, 95% CI -0.43 to 0.04, Z = -1.64, p = 0.10). These findings contradict with results from two prior meta-analyses on this topic [20,21]. Wang et al. [20] concluded that both montelukast and zafirlukast are effective in the treatment and prevention of CC. However, our results suggest that only zafirlukast demonstrates statistically significant efficacy. Pașca et al. [21] reported that Wang et al. [20] probably miscalculated the pooled RDs from the five studies [13-16,18] included in their meta-analysis and got different results. Pașca et al. [21] also reported that montelukast, but not zafirlukast, was effective (six studies were included in their meta-analysis [13-16,18,19]), which also contrasts our findings. This is because of a difference in the method of calculations. While noting the number of positive events in treated and untreated breasts in the study by Graf et al. [13], Pașca et al. [21] reported the number of treated breasts as 74 (37 patients) and 7 positive events. However, when they reported non-treated breasts, they counted them as 45 rather than 90. There were 19 positive events in 45 patients (90 breasts), and not in 45 breasts. This might have led to their current findings. Although all individual studies within the montelukast subgroup reported clinical improvement, the present review finds no statistically significant benefit. Conversely, our findings are consistent with those of Bresnick [14], Reid et al. [16], and Scuderi et al. [18], all of which concluded that zafirlukast is effective in the prevention and treatment of CC.

The dose of LRAs was the same in all studies included in this review, but the duration varied. Montelukast was given 10 mg orally once daily for 3-6 months, whereas the dose of zafirlukast was 20 mg orally twice daily for 3-6 months. The duration varied based upon treatment response and desired reduction in CC grade, patient compliance, side effects, and the safety profile of the drug being used. It may vary as per the intent of therapy. For prevention, the therapy may be given for three months only, but for treatment, the duration may be longer than three months depending upon factors mentioned above. No standardized treatment duration protocol exists as of now.

Although LRAs are generally well-tolerated, however, the use of LRAs is not without complications and side effects. Both the main drugs in the group have some common and distinct adverse effects. Montelukast causes flu-like symptoms, headache, dyspepsia, abdominal pain, and cough [13]. Zafirlukast more commonly causes nausea and headache. Liver failure was also reported in one study [18]. Data from Gryskiewicz [22] raises safety concerns because he reported 66 cases of hepatitis or liver failure from the use of zafirlukast, with 23 deaths. Contraindications for the use of LRAs are deranged liver enzymes or liver functions, pregnancy, and hypersensitivity to LRAs [13]. It is important to note that the use of LRAs for CC is off-label. Patients who are considered fit for the treatment of CC with LRAs should be counseled for these potential adverse effects before the commencement of therapy with a risk-benefit analysis. They should also be counseled to have their liver function tests (LFTs) monitored regularly for the duration of treatment [20].

Based on the findings of this systematic review and current clinical knowledge, the following recommendations can be made: (1) LRAs may be utilized for the prevention and treatment of CC; however, they should be employed as part of a multifactorial strategy that includes strict adherence to aseptic techniques, meticulous surgical methods, and appropriate implant selection and positioning. (2) Zafirlukast appears to be more effective than montelukast in the management of CC and may be considered the agent of choice, unless contraindicated. Given the potential for hepatotoxicity, regular monitoring of LFTs is recommended during zafirlukast therapy. (3) Before considering surgical intervention for CC, patients should be offered a trial of LRA therapy, unless contraindications exist or the patient declines medical management. (4) For patients identified as being at increased risk of developing CC, routine prophylactic use of LRAs may be beneficial and should be considered as part of the postoperative management plan. (5) There is a need for larger, high-quality, and standardized studies, including RCTs, to establish clear protocols and evidence-based guidelines for the use of LRAs in CC management. (6) Patients who are not receiving LRAs for prophylaxis should undergo regular follow-up, allowing for the early detection and treatment of CC at lower Baker grades, when outcomes are likely to be more favorable.

Limitations

This review has several notable limitations. This included only a limited number of studies. Only seven studies met the eligibility criteria for inclusion. None of these were RCTs, and only three were prospective in design. Among the remaining studies, two were observational, and just one was a non-randomized trial [19]. Furthermore, that study was published as a “viewpoint” rather than a standard original article, although it was included due to the relevance and availability of outcome data. The sample sizes in most included studies were relatively small, which may limit the generalizability of the findings, except for the study by Bresnick [14], which included data from 2,499 breasts. Despite good scores on NOS, high heterogeneity remained an issue. The cause of this pooled high heterogeneity might be the array of different procedures, varied implants and their positions, incision types, implant sizes, non-standardized surgical techniques, and many variable patient factors. The literature search was limited to PubMed, including Medline and PubMed Central (PMC), which may have excluded relevant studies indexed in other databases. Only English-language publications were included. This restriction may have led to the exclusion of high-quality studies published in other languages, introducing potential language bias. The inclusion criteria requiring the use of Baker scale excluded two potentially relevant studies. Furthermore, Baker scale, though widely used, is a subjective grading system and may introduce bias due to interobserver variability.

## Conclusions

This systematic review and meta-analysis demonstrate that among the two primary LRAs (montelukast and zafirlukast), zafirlukast shows statistically significant efficacy in the prevention and treatment of CC following implant-based breast surgery. However, to establish the role of LRAs in CC management more definitively, well-designed, high-quality RCTs are needed. Future research should aim to determine optimal treatment duration, assess long-term safety and efficacy, and develop standardized protocols for both prophylactic and therapeutic use of LRAs in diverse patient populations undergoing implant-based breast procedures.

## References

[1] Safran T, Nepon H, Chu CK (2021). Current concepts in capsular contracture: pathophysiology, prevention, and management. Semin Plast Surg.

[2] El-Sheikh Y, Tutino R, Knight C, Farrokhyar F, Hynes N (2008). Incidence of capsular contracture in silicone versus saline cosmetic augmentation mammoplasty: a meta-analysis. Can J Plast Surg.

[3] Cunningham B (2007). The mentor core study on silicone MemoryGel breast implants. Plast Reconstr Surg.

[4] Spear SL, Onyewu C (2000). Staged breast reconstruction with saline-filled implants in the irradiated breast: recent trends and therapeutic implications. Plast Reconstr Surg.

[5] Ho AL, Bovill ES, Macadam SA, Tyldesley S, Giang J, Lennox PA (2014). Postmastectomy radiation therapy after immediate two-stage tissue expander/implant breast reconstruction: a University of British Columbia perspective. Plast Reconstr Surg.

[6] Zino Alarki SM, Mortada H, I Abdullah AI, Alkhalidi H, Alrehaili M (2022). Early onset of capsular contracture after breast augmentation with implant: report of two cases & review of literature. Case Reports Plast Surg Hand Surg.

[7] Tadiparthi S, Staley H, Collis N, O'Donoghue JM (2013). An analysis of the motivating and risk factors for conversion from implant-based to total autologous breast reconstruction. Plast Reconstr Surg.

[8] Swanson E (2016). Open capsulotomy: an effective but overlooked treatment for capsular contracture after breast augmentation. Plast Reconstr Surg Glob Open.

[9] Wolfram D, Rainer C, Niederegger H, Piza H, Wick G (2004). Cellular and molecular composition of fibrous capsules formed around silicone breast implants with special focus on local immune reactions. J Autoimmun.

[10] Polo M, Smith PD, Kim YJ, Wang X, Ko F, Robson MC (1999). Effect of TGF-beta2 on proliferative scar fibroblast cell kinetics. Ann Plast Surg.

[11] Spear SL, Baker JL Jr (1995). Classification of capsular contracture after prosthetic breast reconstruction. Plast Reconstr Surg.

[12] Wells GA, Shea B, O'Connell D, Peterson J, Welch V, Tugwell P (2000). The Newcastle-Ottawa Scale (NOS) for assessing the quality of nonrandomised studies in meta-analyses. 3rd Symposium on Systematic Reviews: Beyond the Basics.

[13] Graf R, Ascenço AS, Freitas RD (2015). Prevention of capsular contracture using leukotriene antagonists. Plast Reconstr Surg.

[14] Bresnick SD (2017). Prophylactic leukotriene inhibitor therapy for the reduction of capsular contracture in primary silicone breast augmentation: experience with over 1100 cases. Plast Reconstr Surg.

[15] Huang CK, Handel N (2010). Effects of Singulair (montelukast) treatment for capsular contracture. Aesthet Surg J.

[16] Reid RR, Greve SD, Casas LA (2005). The effect of zafirlukast (Accolate) on early capsular contracture in the primary augmentation patient: a pilot study. Aesthet Surg J.

[17] Procikieviez IO, Procikieviez O (2024). Leukotriene inhibitors in the prevention of recurring capsular contracture in secondary breast augmentation. Aesthetic Plast Surg.

[18] Scuderi N, Mazzocchi M, Fioramonti P, Bistoni G (2006). The effects of zafirlukast on capsular contracture: preliminary report. Aesthetic Plast Surg.

[19] Lille S, Jacoby J (2018). The potential benefit of preemptive leukotriene inhibitor treatment to breast augmentation/mastopexy surgery. Plast Reconstr Surg.

[20] Wang Y, Tian J, Liu J (2020). Suppressive effect of leukotriene antagonists on capsular contracture in patients who underwent breast surgery with prosthesis: a meta-analysis. Plast Reconstr Surg.

[21] Pașca A, Bonci EA, Chiuzan C (2022). Treatment and prevention of periprosthetic capsular contracture in breast surgery with prosthesis using leukotriene receptor antagonists: a meta-analysis. Aesthet Surg J.

[22] Gryskiewicz JM (2003). Investigation of accolate and singulair for treatment of capsular contracture yields safety concerns. Aesthet Surg J.

